# High-intensity cannabis use and hospitalization: a prospective cohort study of street-involved youth in Vancouver, Canada

**DOI:** 10.1186/s12954-021-00501-8

**Published:** 2021-05-17

**Authors:** Hudson Reddon, M.-J. Milloy, Evan Wood, Ekaterina Nosova, Thomas Kerr, Kora DeBeck

**Affiliations:** 1 BC Centre on Substance Use, University of British Columbia, 400-1045 Howe St, Vancouver, BC V6Z 2A9 Canada; 2 CIHR Canadian HIV Trials Network, 588-1081 Burrard Street, Vancouver, BC V6B 3E6 Canada; 3 Department of Medicine, University of British Columbia, St. Paul’s Hospital, 608-1081 Burrard Street, ,Vancouver, BC V6Z 1Y6 Canada; 4 School of Public Policy, SFU Harbour Centre, Simon Fraser University, 515 West Hastings Street, Vancouver, BC V6B 5K3 Canada

**Keywords:** Cannabis, Hospitalization, Illicit drug use, At-risk youth, Prospective cohort study

## Abstract

**Background:**

There is concern that cannabis use negatively affects vulnerable groups such as youth; however, the relationship between cannabis use and health care utilization has not been well characterized in this population. We longitudinally evaluated the association between daily cannabis use and hospitalization among a prospective cohort of street-involved youth.

**Methods:**

Data were collected from the At-Risk Youth Study (ARYS) in Vancouver, Canada, from September 2005 to May 2015. Participants were interviewed semi-annually and multivariable generalized estimating equation (GEE) logistic regression was used to examine the relationship between daily cannabis use and hospitalization.

**Results:**

A total of 1216 participants (31.2% female) were included in this analysis, and 373 (30.7%) individuals reported hospitalization at some point during the study period. In a multivariable GEE analysis, daily cannabis use was not significantly associated with hospitalization (Adjusted Odds Ratio [AOR] = 1.17, 95% Confidence interval [CI] = 0.84, 1.65). We did observe a significant interaction between daily cannabis use and sex (AOR = 0.51, 95% CI = 0.34, 0.77), whereby cannabis use was associated with a decreased odds of hospitalization among males (AOR = 0.60, 95% CI = 0.47, 0.78), yet was not significantly associated with hospitalization among females (AOR = 1.19, 95% CI = 0.84, 1.67).

**Conclusions:**

The finding that daily cannabis use was not associated with hospitalization among street-involved youth is encouraging given the high rates of cannabis use in this population and the expansion of cannabis legalization and regulation. Future studies, however, are warranted to monitor possible changes in the consequences of cannabis use as cannabis legalization and regulation increase internationally.

## Background

Cannabis remains the world’s most widely produced, trafficked and consumed illicit drug and concerns have been raised about the potential harms associated with acute and chronic cannabis use, including problems with cognitive and psychomotor functioning, respiratory diseases, dependence and mental illness [1–4]. Some emerging evidence suggests that adolescents and young adults may be uniquely vulnerable to the long-term effects of intense or chronic cannabis use due to the neurological, social and educational development that occurs during this period [5, 6]. As a result, young adults experiencing acute (e.g., accident-related injuries) or long-term (e.g., dependence, educational attainment) harm from cannabis use may account for a greater overall burden of illness compared to older individuals who experience these outcomes [7–10]. A 2017 analysis showed that among repeated users, the average age of first cannabis use was 18.6 years and more than 50% of cannabis users reported repeated use before the age of 18 [11].

Although several studies have reported on harms associated with cannabis use, work in this area remains controversial and the extent of these harms has been difficult to quantify [12]. High-intensity cannabis use has been linked to a risk of respiratory complications, yet experts have questioned the strength of this association and nearly half of past-year cannabis users also smoke tobacco making it likely that tobacco is either a substantial contributor or primary cause of the association between cannabis use and respiratory diseases [13, 14]. The cognitive and psychomotor impairments of cannabis have also been described although these changes typically dissipate in adults after use has been discontinued for several weeks, and some work suggests that the observed effects might be explained by socioeconomic differences [8, 12]. Even among young people who may be more susceptible to the adverse effects of cannabis use, severe harms only occur in a minority of users and the vast majority does not experience mental illness or dependence [6, 15–17].

Nevertheless, the morbidity and mortality associated with cannabis use remains a public health concern, particularly in countries implementing cannabis legalization policies. Studies from the USA and Europe have reported elevated rates of hospitalization among people who use cannabis and significant increases in cannabis-related hospitalizations were observed throughout the introduction of cannabis legalization in Colorado [18–20]. In Canada, cannabis-attributable lung cancer represents the largest contributor to mortality among people who use cannabis, while cannabis use disorder was the primary contributor to the overall cannabis burden of disease [16]. The burden of disease associated with cannabis also varied by age and sex, with the highest burden occurring among young people and males accounted for twice as much burden as females [16]. Prior studies have demonstrated that men and women differ in the prevalence of cannabis use and cannabis use disorder, drug tolerance, health outcomes and treatment-seeking behavior related to cannabis use [21–23]. Compared to women, men typically; initiate cannabis use at a younger age; have greater access to cannabis; are more likely to use frequently and in higher quantities; are more likely to be long-term users; and are twice as likely to be diagnosed with a cannabis use disorder [24, 25]. However, women have been found to have more severe symptoms associated with cannabis withdrawal such as nausea and anxiety [26, 27]. Among patients hospitalized for injury-related events, men who used cannabis exhibited increased rates of injuries resulting from motor vehicle accidents, assault and self-inflicted injuries compared to non-users [18]. Women who used cannabis were only more likely to sustain self-inflicted injuries compared to non-users [18]. Many large-scale studies have also found that women are significantly more likely than men to access healthcare services including primary and specialty care [28, 29].

A recent study of emergency department access reported significant increases in emergency department visits related to cannabis use, with the largest increase observed among youth [30]. Youth who use drugs and are street-involved (homeless or use homeless youth services) are at an increased risk to initiate high-risk substance use such as injection drug use and engage in high-risk behaviors including binging and needle sharing [31–33]. As a result, they experience increased rates of infectious diseases, mental illness, intentional and unintentional injuries and overdoses [34, 35]. For many of these patients, hospitalization is necessary due to the complications associated with these comorbidities [36]. However, barriers such as transportation, cost, fear of judgment and lack of trust often discourage these youth from accessing health and social services to address these health vulnerabilities [37, 38]. Avoiding services for these health issues often allows them to deteriorate until they require hospitalization, which increases the health and economic burden associated with treating these conditions [35, 37, 39, 40]. Previous studies of street-involved youth have found that nearly one-third report being hospitalized in the last 6 months and many authors have raised concerns about how cannabis use affects the acute and long-term health of vulnerable populations such as economically disadvantaged youth [41, 42].

The risk environment for young people who use drugs, including the social, environmental and structural factors that mediate health outcomes, is changing with the expansion of legal and regulated cannabis throughout North America [43]. Rhodes’ Risk Environment Framework outlines how social, structural and environmental factors shape health-related behaviors and health care service utilization [44]. Among people who use drugs, this framework can be applied to examine the interactions between social and structural factors with individual factors and how these interactions influence high-risk practices and health service access among vulnerable populations [45]. Given that few studies have examined the impact of cannabis use on health care utilization and associated expenditures such as hospitalization, particularly among youth, we sought to longitudinally evaluate if high-intensity cannabis use was associated with hospitalization among a prospective cohort of street-involved youth who use illicit drugs. To account for the sex differences in the prevalence of cannabis use and health outcomes associated with cannabis use, we also examined sex × cannabis use interaction effects to determine if the effect of high-intensity cannabis use on hospitalization was different among males and females.

## Methods

The data for this analysis were collected from the At-Risk Youth Study (ARYS). ARYS is an open ongoing prospective cohort of street-involved youth that has been described in detail previously [46]. Briefly, participants were recruited through snowball sampling and extensive street outreach methods in Vancouver, Canada. Eligibility criteria included: being aged 14–26 years at the time of recruitment; illicit drug use other than or in addition to cannabis in the past 30 days; providing written informed consent; and street-involved, defined as being without stable housing or accessing street-based youth services in the past 6 months [47]. An interviewer-administered questionnaire was completed by participants at baseline and every 6-months thereafter to collect data related to socio-demographic information, substance use behaviors and engagement with health and social services. Participants were remunerated $30 CAD as compensation for each interview, and the ARYS study has been approved by the University of British Columbia’s Research Ethics Board.

This study included all ARYS participants recruited between September 2005 and May 2015. The main outcome was any experience of hospitalization in the past 6 months. This was assessed through self-report based on the item, “Have you been admitted to the hospital in the last 6 months (yes vs. no). Participants who answered affirmatively then specified the specific condition that they were admitted for based on the item, “In the last 6 months, what serious medical condition or conditions were you hospitalized for (endocarditis, cellulitis, abscess, pneumonia, surgery, osteomyelitis, liver failure, mental health issues, infection, drug-related (e.g., overdose, withdrawal), other)?” The primary explanatory variable of interest was daily cannabis use (≥ daily vs. < daily). Cannabis use was measured based on the item, “In the last 6 months, how often have you used marijuana?” The response options include, "0 = less than once a month, 1 = 1–3 times a month, 2 = Once a week, 3 = 2 or more times a week, 4 = at least daily.” These categories were collapsed to ≥ daily vs. < daily due to the low prevalence of occasional users (e.g., “less than once a month” and “1–3 times per month”) and analyzing these as individual categories can produce unstable estimates of effect size.

Additional factors potentially associated with hospitalization were also included in the analysis. These variables included: age (per year older); sex (male vs. female); ethnicity/ancestry (White vs. others); homelessness (yes vs. no); mental illness (yes vs. no), recent injection drug use (yes vs. no); heavy alcohol use (yes vs. no); frequent heroin use (≥ daily vs. < daily); frequent cocaine use (≥ daily vs. < daily); frequent crack use (≥ daily vs. < daily); frequent methamphetamine use (≥ daily vs. < daily); non-fatal overdose (yes vs. no); and any alcohol or drug treatment (yes vs. no). The measurement of mental illness was based on self-report, and defined as any diagnosis of a mental health disorder at baseline or over follow-up. At baseline, this was assessed based on the item “Have you ever been diagnosed with a mental health issue (yes vs. no)?” Over follow-up, this was assessed based on the item, “Have you been diagnosed with a mental health issue in the last 6 months? (yes vs. no).” Participants who self-reported one or more diagnosis including depression, obsessive–compulsive disorder, schizophrenia, post-traumatic stress disorder (PTSD), drug-induced psychosis, bipolar disorder, attention-deficit disorder, attention-deficit hyperactivity disorder, oppositional defiance disorder, personality disorders, sleep disorders or other were classified and having a mental illness. Heavy alcohol use was defined based on the National Institute on Alcohol Abuse and Alcoholism (NIAAA) definition of risky alcohol use: > 14 drinks/ week or > 4 drinks on 1 occasion for men < 65 years of age, and > 7 drinks/week or > 3 drinks on 1 occasion for all women and men ≥ 65 years of age [48]. Each of the covariates, as well as the outcome and primary explanatory variable of interest were time-varying and assessed at baseline and semi-annually through the interviewer-administered questionnaire. All behavioral variables referred to the preceding 6-month period and the variable definitions were based on previous studies [49].

Baseline characteristics of the study sample, stratified by cannabis use in the last 6 months, were analyzed using Cochran–Armitage trend test for categorical variables and Kruskal–Wallis test for continuous variables. Generalized estimating equation (GEE) logistic regression was used for the analysis of correlated follow-up data to identify factors associated with being hospitalized in the past 6-months. GEE were used since this method provides standard errors adjusted for multiple observations for each participant over follow-up using an exchangeable correlation structure and can be applied to participants with varying numbers of observations [50–54]. Before conducting the primary analysis, bivariable GEE analyses were performed to determine the associations between hospitalization and each of the variables of interest. The multivariable model was fit using an a priori-defined statistical protocol based on the quasi-likelihood under the independence model criterion (QIC) for GEE and *p* values [55]. The initial multivariable model included all explanatory variables associated with hospitalization in the bivariable analyses at the level of *p* < 0.10. Reduced models were built by sequentially removing each variable with the highest *p* value, and the final model included the set of variables associated with the lowest quasi-likelihood under the independence model criterion (QIC). Given the sex differences in the prevalence of cannabis use, we also included a sex × cannabis interaction term to determine if the effect of cannabis use on hospitalization was different among males and females [56]. All statistical analyses were conducted using R version 3.2.4 (R Core Team, Vienna, Austria). All *p* values are two sided.

## Results

Between September 2005 and May 2015, a total of 1216 participants completed at least one follow-up visit and were included in the present study: 379 (31.2%) were female and the median age at baseline was 21.8 years (interquartile range [IQR] 19.8–23.6 years). The median number of study visits completed by the participants was three (IQR = 1–5) and the median observation time per participant was 17.1 months (IQR = 0–32.6). At baseline, 530 (43.6%) participants reported at least daily cannabis use in the last 6 months and 151 (12.4%) individuals reported experiencing a hospitalization in the last 6 months. The baseline characteristics of the study sample stratified by cannabis use in the last 6 months are shown in Table 1.

**Table 1:** Baseline characteristics among street-involved youth who use illicit drugs in Vancouver, Canada (*n* = 1216)

	Daily cannabis use in the past 6 months		
	No (*n* = 682) *n* (%)	Yes (*n* = 530) *n* (%)	*p* value
*Age (per additional year)*			
Median (IQR)	21.9 (19.9–23.7)	21.8 (19.7–23.6)	0.502
*White ethnicity*			
Yes	454 (66.6)	362 (68.3)	0.539
No	226 (33.1)	167 (31.5)	
*Sex*			
Male	412 (60.4)	421 (79.4)	**< 0.001**
Female	270 (39.6)	109 (20.6)	
*Homeless*^a^			
Yes	503 (73.8)	397 (74.9)	0.777
No	175 (25.7)	133 (25.1)	
*Mental illness*			
Yes	352 (51.6)	278 (52.5)	0.772
No	330 (48.4)	252 (47.5)	
*Injection drug use*^a^			
Yes	265 (38.9)	137 (25.8)	**< 0.001**
No	416 (61.0)	392 (74.0)	
*Heavy alcohol use*			
Yes	234 (34.3)	198 (37.4)	0.286
No	445 (65.2)	331 (62.5)	
*Hospitalization*^a^			
Yes	101 (14.8)	50 (9.4)	**0.005**
No	581 (85.2)	480 (90.6)	
*Frequent heroin use*^a^			
≥ Daily	99 (14.5)	37 (7.0)	**< 0.001**
< Daily	576 (84.5)	486 (91.7)	
*Frequent cocaine use*^a^			
≥ Daily	20 (2.9)	17 (3.2)	0.790
< Daily	656 (96.2)	510 (96.2)	
*Frequent crack use*^a^			
≥ Daily	103 (15.1)	80 (15.1)	0.965
< Daily	577 (84.6)	445 (84.0)	
*Frequent methamphetamine use*^a^			
≥ Daily	92 (13.5)	70 (13.2)	0.889
< Daily	584 (85.6)	455 (85.8)	
*Non-fatal overdose*^a^			
Yes	95 (13.9)	75 (14.2)	0.908
No	585 (85.8)	453 (85.5)	
*Drug or alcohol treatment*^a^			
Yes	227 (33.3)	146 (27.5)	**0.031**
No	448 (65.7)	379 (71.5)	

Bold values indicate *p* values < 0.05

^a^Refers to activities in the 6 months prior to follow-up interview, not all cells add up to 1216 as participants may choose not to answer certain questions

As indicated in Table 2, the bivariable analyses revealed that factors positively associated with hospitalization included: homelessness (odds ratio [OR] = 1.41; 95% confidence interval [CI]: 1.18, 1.68); mental illness (OR = 1.87; 95% CI 1.50, 2.35); daily cocaine use (OR = 2.35; 95% CI 1.50, 3.70); non-fatal overdose (OR = 1.98; 95% CI 1.55, 2.51); and participation in alcohol or drug treatment (OR = 1.58; 95% CI 1.33, 1.89). Daily cannabis use was the only factor negatively associated with hospitalization in the bivariable analysis (OR = 0.70; 95% CI 0.58, 0.85).

**Table 2:** Bivariable and multivariable GEE analysis of factors associated with hospitalization (*n* = 1216)

Characteristic	Unadjusted		Adjusted		Subgroup analysis	
	Odds ratio (95% CI)	*p* value	Odds ratio (95% CI)	*p* value	Odds ratio (95% CI)	*p* value
*Frequent cannabis use*^a^						
(≥ Daily vs. < daily)	0.70 (0.58–0.85)	**< 0.001**	1.17 (0.84–1.65)	0.359		
Frequent cannabis use^a^ × Sex interaction	–	–	0.51 (0.34–0.77)	**0.002**		
	Frequent cannabis use among females				1.19 (0.84–1.67)	0.327
	Frequent cannabis use among males				0.60 (0.47–0.78)	**< 0.001**
*Age*						
(Per additional year older)	1.00 (0.97–1.03)	0.773				
*White ethnicity*						
(Yes vs. no)	1.05 (0.82–1.34)	0.703				
*Sex*						
(Female vs. male)	0.90 (0.72–1.13)	0.363	1.17 (0.89–1.55)	0.255		
*Homeless*^a^						
(yes vs. no)	1.41 (1.18–1.68)	**< 0.001**	1.44 (1.18–1.76)	**< 0.001**		
*Mental illness*						
(Yes vs. no)	1.87 (1.50–2.35)	**< 0.001**	1.74 (1.38–2.20)	**< 0.001**		
*Injection drug use*^a^						
(yes vs. no)	1.29 (1.05–1.57)	0.013				
*Heavy alcohol use*						
(Yes vs. no)	0.97 (0.78–1.20)	0.766				
*Frequent heroin use*^a^						
(≥ Daily vs. < daily)	1.11 (0.87–1.41)	0.396				
*Frequent cocaine use*^a^						
(≥ daily vs. < daily)	2.35 (1.50–3.70)	**< 0.001**	2.02 (1.28–3.20)	**0.003**		
*Frequent crack use*^a^						
(≥ Daily vs. < daily)	1.13 (0.85–1.50)	0.417				
*Frequent methamphetamine use*^a^						
(≥ Daily vs. < daily)	1.13 (0.88–1.45)	0.340				
*Non-fatal overdose*^a^						
(Yes vs. no)	1.98 (1.55–2.51)	**< 0.001**	1.73 (1.34–2.22)	**< 0.001**		
*Drug or alcohol treatment*^a^						
(yes vs. no)	1.58 (1.33–1.89)	**< 0.001**	1.45 (1.21–1.74)	**< 0.001**		

Bold values indicate *p* values < 0.05

^a^Refers to activities in the 6 months prior to follow-up interview

The results of the multivariable analysis of factors associated with hospitalization are also presented in Table 2. Factors that remained positively associated with hospitalization included: homelessness (AOR = 1.44; 95% CI 1.18, 1.76); mental illness (AOR = 1.74; 95% CI 1.38, 2.20); daily cocaine use (AOR = 2.02; 95% CI 1.28, 3.20); non-fatal overdose (AOR = 1.73; 95% CI 1.34, 2.22); and participation in alcohol or drug treatment (AOR = 1.45; 95% CI 1.21, 1.74). Daily cannabis use was not significantly associated with hospitalization in the multivariable model (AOR = 1.17; 95% CI 0.84, 1.65). We observed a significant interaction between daily cannabis use and sex (AOR = 0.51; 95% CI 0.34, 0.77), whereby daily cannabis use was not associated with hospitalization among females (AOR = 1.19; 95% CI 0.84, 1.67) but was negatively associated with hospitalization among males (AOR = 0.60; 95% CI 0.47, 0.78). This indicates that the odds of hospitalization are decreased by 40% (1–0.6 = 0.4) among males compared to females, and that the probability of hospitalization associated with being male is 37.5% (p = OR / (1 + OR)).

## Discussion

In this prospective cohort study, we observed a high prevalence of daily cannabis use among a community-recruited sample of street-involved youth in Vancouver, Canada. In our multivariable analysis, daily cannabis use was not significantly associated with recent hospitalization. Although, we observed a significant interaction between sex and cannabis use whereby daily cannabis use was not significantly associated with hospitalization among females, yet was negatively associated with hospitalization among males. Consistent with the Risk Environment Framework, both individual and structural factors were associated with hospitalization, including homelessness, frequent cocaine use, experiencing a non-fatal overdose and participation in alcohol or drug treatment. These associations were observed after adjustment for a range of possible socio-demographic and drug use confounders.

There is evidence to explain the lack of association between cannabis use and hospitalization. Specifically, people who use cannabis may reflect a subgroup of people who use drugs that are averse to the risks associated with other illicit drug use and injection drug use, such as the perceived risks of addiction, dependence and interference with life goals [57, 58]. However, this explanation may not apply since we adjusted for the use of a number of high-risk drugs in the multivariable analysis. There is also emerging evidence to suggest that cannabis may be used by some individuals to intentionally reduce the use of “high-risk substances” including crack-cocaine and opioids [59, 60]. Cannabis has been used intentionally and effectively to reduce cocaine-related craving symptoms and the use of crack-cocaine among people who use illicit drugs [61]. Frequent cannabis use has also been associated with decreased illicit opioid use and exposure to fentanyl among people who use drugs [62, 63]. Previous studies have also found that cannabis was often intentionally used as a method to “self-detox,” decrease the severity of their addiction, transition to less dangerous routes of substance administration and to maintain low-intensity heroin use or transition off of heroin [64, 65]. Cannabinoid receptors operate in part through an opioid receptor mechanism and increase the concentrations of dopamine in the nucleus accumbens, similar to heroin and other commonly used opioids [66–68]. Existing studies have demonstrated independent analgesic effects of cannabis and improved analgesia when opioids are supplemented with a cannabinoid CB1 agonist [69, 70]. Since the use of crack-cocaine and licit and illicit opioids has been linked to health and social harms including increased risk of HIV, hepatitis C virus, comorbid mental illness, and overdose, decreased hospitalization among people who use cannabis may be an indirect effect of cannabis users reducing the use of high-risk substances [71–73].

Another explanation for the lack of association between cannabis use and hospitalization may involve the type of harm caused by cannabis. Cannabis attributed lung cancer, cannabis use disorder and motor vehicle accidents are the primary sources of disease burden associated with cannabis use [16]. Since cannabis associated lung cancer and cannabis use disorder are more likely to affect older individuals with a long history of chronic cannabis use, it is possible that these harms were not yet detectable in our cohort. In addition, some authors have challenged the link between cannabis use and lung cancer, and a previous systematic review failed to identify a significant association between cannabis use and lung cancer after adjustment for tobacco use [14]. Many of these participants are also experiencing financial hardship and do not have access to motor vehicles, which may have reduced the prevalence of driving under the influence of cannabis. Acute paranoia and psychosis associated with poly-substance use is another potential source of hospitalization, yet the relative safety of cannabis and the other risks of hospitalization among this cohort may explain the non-significant association we observed [16, 74].

The cannabis use × sex interaction we observed indicated that daily cannabis use was associated with decreased hospitalization among males, yet was not significantly associated with hospitalization among females. It is possible that youth who use drugs, particularly males, avoid accessing health services due to the fear of stigma associated with their substance use [75]. Men are significantly less likely than women to seek help for both medical and psychological health issues, and the stigma associated with illicit drug use may compound the avoidance of health care services among young male cannabis users [75, 76].

As suggested by the Risk Environment Framework, both individual and structural factors were significantly associated with hospitalization. Consistent with previous studies, substance use related factors including frequent cocaine use and non-fatal overdose were associated with an increased risk of hospitalization in this sample [41]. Due to the short half-life of cocaine (40–60 min), cocaine users often use over 20 times per day and are an increased risk of cardiovascular complications, psychotic episodes and acquiring blood-borne viral infections, particularly among people who inject drugs [77–79]. Among people who use drugs, homelessness has been associated with public drug use, bacterial infections, needle sharing, overdose risk and hospitalization [80–82]. These results support the importance of individual and structural exposures in shaping health-related behaviors and healthcare utilization among young people who use drugs.

Strengths of this study include the prospective cohort design that performed repeated measures semi-annually. We also included a range of socio-demographic and drug use variables to provide a comprehensive analysis of risk factors for hospitalization. Through this method we were able to analyze multiple independent risk factors for hospitalization that were time-varying over the study period. Limitations of this study include collecting drug use and hospitalization measures through self-report, which introduced the potential for socially desirable reporting of stigmatized behaviors and recall error. It is also possible that the impact of these biases may have changed over the study period. Since the expansion of medical and recreational cannabis use policies has been linked to decreases in the stigmatization of cannabis use behaviors, cannabis use may have been measured more accurately in recent follow-up visits compared to measures earlier in the study period [83]. If cannabis use did increase the risk of hospitalization and was underreported in earlier follow-up visits, this may have attenuated the association we observed between cannabis use and hospitalization. However, assessing these variables through self-report has been performed in previous studies and shown to provide valid and reliable measurements [41, 84, 85]. It is also possible that measuring mental health disorders through self-report of previous diagnoses may have introduced recall error or bias, and undiagnosed mental health disorders were not captured in this assessment. People who use drugs and are living with mental health disorders are less likely to access health and social services and frequently experience stigma and discrimination in healthcare settings [86]. Self-medication of psychiatric symptoms with drugs and alcohol is also common among people living with mental health disorders and is associated with poorer health outcomes [87]. The potential harms associated with cannabis use among individuals with mental health conditions may have been underestimated in the present study if undiagnosed mental health disorders prohibited these participants from accessing health services such as hospitals. Including hospitalization events that are not attributable to cannabis use may have contributed to the lack of association observed between cannabis use and hospitalization. Since data for the frequency of cannabis use per day were not available, we were not able to differentiate people who used once per day from people who used multiple times per day. This may have prevented our measure of cannabis use frequency from isolating the most frequent cannabis users who may be at an increased risk to experience hospitalization. Assessing cannabis use and hospitalization at the same follow-up visit also creates uncertainty about the temporal relationship between cannabis use and hospitalization. It is possible that the direction of this association may be reversed if participants who were hospitalized were more likely to subsequently decrease their cannabis use. In addition, ARYS is not a random sample and given that ARYS is comprised of street-involved youth who use unregulated drugs in addition to cannabis, these results may not be generalizable to young people who use cannabis and do not use other drugs. The use of other high-risk substances (e.g., cocaine, crystal methamphetamine) among our study sample is associated with an increased risk of hospitalization and the additional use of cannabis may not significantly increase the risk of hospitalization among people concurrently using other unregulated substances [41, 88]. Therefore, the association between cannabis use and hospitalization may be different among youth who use cannabis and do not also use high-risk unregulated substances. Although few participants died during the study (N = 14) and the distribution of these deaths did not vary by cannabis use (P = 0.781), it is possible that the association between cannabis use and health conditions requiring hospitalization may be different among older cannabis users who are more likely to experience cannabis-related harm associated with chronic use. Lastly, the potential for residual confounding to impact the association between daily cannabis use and hospitalization is a concern due to the observational study design.

## Conclusions

In the present study, we analyzed the association between daily cannabis use and hospitalization among a prospective cohort of street-involved youth. While daily cannabis use was not significantly associated with hospitalization in the whole sample, we observed sex-specific effects for the impact of cannabis use: daily cannabis use was associated with decreased rates of hospitalization among males and was not significantly associated with hospitalization among females. Given that a number of countries internationally are exploring novel approaches to the regulation of recreational cannabis, future research is required to characterize the health and social consequences of cannabis use and determine how the regulatory frameworks influence these outcomes and associated healthcare expenditures among the general population.

## Data Availability

The datasets used and/or analyzed during the current study are available from the corresponding author on a reasonable request.

## References

[1] Kalant H (2004). Adverse effects of cannabis on health: an update of the literature since 1996. Prog Neuropsychopharmacol Biol Psychiatry.

[2] Crepault JF (2014). Cannabis policy framework.

[3] National Academies of Sciences, Engineering, and Medicine. Washington, DC: The National Academies Press: The health effects of cannabis and cannabinoids: Current state of evidence and recommendations for research; 2017. 28182367

[4] UNODC. World Drug Report 2016. Vienna: United Nations Office on Drugs and Crime; 2016.

[5] Silins E, Horwood LJ, Patton GC, Fergusson DM, Olsson CA, Hutchinson DM (2014). Young adult sequelae of adolescent cannabis use: an integrative analysis. The Lancet Psychiatry.

[6] Squeglia LM, Jacobus J, Tapert SF (2009). The influence of substance use on adolescent brain development. Clin EEG Neurosci.

[7] Degenhardt L, Ferrari AJ, Calabria B, Hall WD, Norman RE, McGrath J (2013). The global epidemiology and contribution of cannabis use and dependence to the global burden of disease: results from the GBD 2010 study. PLoS ONE.

[8] Block RI, O'Leary DS, Hichwa RD, Augustinack JC, Boles Ponto LL, Ghoneim MM (2002). Effects of frequent marijuana use on memory-related regional cerebral blood flow. Pharmacol Biochem Behav.

[9] The Health Effects of Cannabis and Cannabinoids: The Current State of Evidence and Recommendations for Research. The National Academies Collection: Reports funded by National Institutes of Health. Washington (DC): National Academies Press (US) (2017). 28182367

[10] Wettlaufer A, Florica RO, Asbridge M, Beirness D, Brubacher J, Callaghan R (2017). Estimating the harms and costs of cannabis-attributable collisions in the Canadian provinces. Drug Alcohol Depend.

[11] Statistics Canada. Ottawa: Canadian Tobacco, Alcohol and Drugs Survey, 2017; 2018.

[12] Rogeberg O (2013). Correlations between cannabis use and IQ change in the Dunedin cohort are consistent with confounding from socioeconomic status. Proc Natl Acad Sci USA.

[13] Fischer B, Jeffries V, Hall W, Room R, Goldner E, Rehm J. Lower Risk Cannabis use Guidelines for Canada (LRCUG): a narrative review of evidence and recommendations. Canadian journal of public health = Revue canadienne de sante publique. 2011;102(5):324–7. 10.1007/BF03404169 PMC6973752 22032094

[14] Mehra R, Moore BA, Crothers K, Tetrault J, Fiellin DA (2006). The association between marijuana smoking and lung cancer: a systematic review. Arch Intern Med.

[15] Degenhardt L, Hall WD, Lynskey M, McGrath J, McLaren J, Calabria B, et al. Should burden of disease estimates include cannabis use as a risk factor for psychosis? PLoS medicine. 2009;6(9):e1000133. 10.1371/journal.pmed.1000133 PMC2741573 19787023

[16] Imtiaz S, Shield KD, Roerecke M, Cheng J, Popova S, Kurdyak P (2016). The burden of disease attributable to cannabis use in Canada in 2012. Addiction.

[17] Fischer B, Rehm J, Crepault JF (2016). Realistically furthering the goals of public health by cannabis legalization with strict regulation: Response to Kalant. Int J Drug Policy.

[18] Gerberich SG, Sidney S, Braun BL, Tekawa IS, Tolan KK, Quesenberry CP (2003). Marijuana use and injury events resulting in hospitalization. Ann Epidemiol.

[19] Jouanjus E, Leymarie F, Tubery M, Lapeyre-Mestre M (2011). Cannabis-related hospitalizations: unexpected serious events identified through hospital databases. Br J Clin Pharmacol.

[20] Kim HS, Monte AA (2016). Colorado Cannabis Legalization and Its Effect on Emergency Care. Ann Emerg Med.

[21] Cotto JH, Davis E, Dowling GJ, Elcano JC, Staton AB, Weiss SR (2010). Gender effects on drug use, abuse, and dependence: a special analysis of results from the National Survey on Drug Use and Health. Gend Med.

[22] Khan SS, Secades-Villa R, Okuda M, Wang S, Perez-Fuentes G, Kerridge BT (2013). Gender differences in cannabis use disorders: results from the National Epidemiologic Survey of Alcohol and Related Conditions. Drug Alcohol Depend.

[23] Ketcherside A, Baine J, Filbey F (2016). Sex Effects of Marijuana on Brain Structure and Function. Curr Addict Rep.

[24] Wagner FA, Anthony JC (2002). Into the world of illegal drug use: exposure opportunity and other mechanisms linking the use of alcohol, tobacco, marijuana, and cocaine. Am J Epidemiol.

[25] Zhu H, Wu LT (2017). Sex differences in cannabis use disorder diagnosis involved hospitalizations in the United States. J Addict Med.

[26] Schlienz NJ, Budney AJ, Lee DC, Vandrey R (2017). Cannabis Withdrawal: A Review of Neurobiological Mechanisms and Sex Differences. Curr Addict Rep.

[27] Cuttler C, Mischley LK, Sexton M (2016). Sex Differences in Cannabis Use and Effects: A Cross-Sectional Survey of Cannabis Users. Cannabis and cannabinoid research.

[28] Hippisley-Cox J, Vinogradova Y. Trend in consultation rates in general practice 1995 to 2008: analysis of the QRESEARCH database. NHS Information Centre. 2009.

[29] Wang Y, Hunt K, Nazareth I, Freemantle N, Petersen I (2013). Do men consult less than women? An analysis of routinely collected UK general practice data. BMJ Open.

[30] Zhu H, Wu LT (2016). Trends and correlates of cannabis-involved emergency department visits: 2004 to 2011. J Addict Med.

[31] Reddon H, DeBeck K, Socias ME, Dong H, Wood E, Montaner J (2018). Cannabis use is associated with lower rates of initiation of injection drug use among street-involved youth: A longitudinal analysis. Drug Alcohol Rev.

[32] Lloyd-Smith E, Kerr T, Zhang R, Montaner J, Wood E (2008). High prevalence of syringe sharing among street involved youth. Addiction Research Theory.

[33] Mitra G, Wood E, Nguyen P, Kerr T, DeBeck K (2015). Drug use patterns predict risk of non-fatal overdose among street-involved youth in a Canadian setting. Drug Alcohol Depend.

[34] Marshall BD, Grafstein E, Buxton JA, Qi J, Wood E, Shoveller JA (2012). Frequent methamphetamine injection predicts emergency department utilization among street-involved youth. Public Health.

[35] Edidin JP, Ganim Z, Hunter SJ, Karnik NS (2012). The mental and physical health of homeless youth: a literature review. Child Psychiatry Hum Dev.

[36] Wu LT, Zhu H, Ghitza UE (2018). Multicomorbidity of chronic diseases and substance use disorders and their association with hospitalization: Results from electronic health records data. Drug Alcohol Depend.

[37] Chelvakumar G, Ford N, Kapa HM, Lange HLH, McRee AL, Bonny AE (2017). Healthcare Barriers and Utilization Among Adolescents and Young Adults Accessing Services for Homeless and Runaway Youth. J Community Health.

[38] Ensign J, Bell M (2004). Illness experiences of homeless youth. Qual Health Res.

[39] Ensign J, Gittelsohn J. Health and access to care: perspectives of homeless youth in Baltimore City, U.S.A. Social science & medicine. 1998;47(12):2087–99. 10.1016/s0277-9536(98)00273-1 10075249

[40] Salit SA, Kuhn EM, Hartz AJ, Vu JM, Mosso AL (1998). Hospitalization costs associated with homelessness in New York City. N Engl J Med.

[41] Chang DC, Rieb L, Nosova E, Liu Y, Kerr T, DeBeck K (2018). Hospitalization among street-involved youth who use illicit drugs in Vancouver, Canada: a longitudinal analysis. Harm Reduct J.

[42] Windle SB, Wade K, Filion KB, Kimmelman J, Thombs BD, Eisenberg MJ. Potential harms from legalization of recreational cannabis use in Canada. Canadian journal of public health = Revue canadienne de sante publique. 2019. 10.17269/s41997-018-00173-1 PMC6964625 30759307

[43] Cannabis Legalization and Regulation. Government of Canada: Department of Justice; 2018.

[44] Rhodes T (2002). The, “risk environment”: a framework for understanding and reducing drug-related harm. International Journal of Drug Policy.

[45] Rhodes T, Singer M, Bourgois P, Friedman SR, Strathdee SA (2005). The social structural production of HIV risk among injecting drug users. Soc Sci Med.

[46] Wood E, Stoltz JA, Montaner JS, Kerr T (2006). Evaluating methamphetamine use and risks of injection initiation among street youth: the ARYS study. Harm Reduct J.

[47] Boivin JF, Roy E, Haley N, Galbaud du Fort G. The health of street youth: a Canadian perspective. Can J Public Health = Revue canadienne de sante publique. 2005;96(6):432–7. 10.1007/BF03405183 PMC6975913 16350867

[48] Willenbring ML, Massey SH, Gardner MB (2009). Helping patients who drink too much: an evidence-based guide for primary care clinicians. Am Fam Physician.

[49] Wood E, Tyndall MW, Spittal PM, Li K, Kerr T, Hogg RS (2001). Unsafe injection practices in a cohort of injection drug users in Vancouver: Could safer injecting rooms help?. Can Med Assoc J.

[50] Hubbard AE, Ahern J, Fleischer NL, Van der Laan M, Lippman SA, Jewell N (2010). To GEE or not to GEE: comparing population average and mixed models for estimating the associations between neighborhood risk factors and health. Epidemiology.

[51] Locascio JJ, Atri A (2011). An overview of longitudinal data analysis methods for neurological research. Dementia and geriatric cognitive disorders extra.

[52] Stokes ME. Recent advances in categorical data analysis. 24th Annual Meeting of the SAS Users Group International. SAS Institute Inc; Cary, North Carolina, USA1999.

[53] Wang L (2011). GEE analysis of clustered binary data with diverging number of covariates. Ann Stat.

[54] Fitzmaurice G, Laird N, Ware J (2011). Applied longitudinal analysis.

[55] Pan W (2001). Akaike's information criterion in generalized estimating equations. Biometrics.

[56] Rotermann M, Langlois K (2012). Prevalence and correlates of marijuana use in Canada, 2012. Health Rep.

[57] Hadland SE, Kerr T, Marshall BD, Small W, Lai C, Montaner JS (2010). Non-injection drug use patterns and history of injection among street youth. Eur Addict Res.

[58] Roy E, Nonn E, Haley N (2008). Transition to injection drug use among street youth–a qualitative analysis. Drug Alcohol Depend.

[59] Socias ME, Kerr T, Wood E, Dong H, Lake S, Hayashi K (2017). Intentional cannabis use to reduce crack cocaine use in a Canadian setting: A longitudinal analysis. Addict Behav.

[60] Kral AH, Wenger L, Novak SP, Chu D, Corsi KF, Coffa D (2015). Is cannabis use associated with less opioid use among people who inject drugs?. Drug Alcohol Depend.

[61] Dreher M (2002). Crack heads and roots daughters: The therapeutic use of cannabis in Jamaica. Journal of Cannabis Therapeutics.

[62] Lake S, Walsh Z, Kerr T, Cooper ZD, Buxton J, Wood E, et al. Frequency of cannabis and illicit opioid use among people who use drugs and report chronic pain: A longitudinal analysis. PLoS medicine. 2019;16(11):e1002967. 10.1371/journal.pmed.1002967 PMC6863529 31743343

[63] Hayashi K, Milloy MJ, Lysyshyn M, DeBeck K, Nosova E, Wood E (2018). Substance use patterns associated with recent exposure to fentanyl among people who inject drugs in Vancouver, Canada: A cross-sectional urine toxicology screening study. Drug Alcohol Depend.

[64] Bozinoff N, Small W, Long C, DeBeck K, Fast D (2017). Still, "at risk": An examination of how street-involved young people understand, experience, and engage with "harm reduction" in Vancouver's inner city. Int J Drug Policy.

[65] Wenger LD, Lopez AM, Comfort M, Kral AH (2014). The phenomenon of low-frequency heroin injection among street-based urban poor: drug user strategies and contexts of use. Int J Drug Policy.

[66] Tanda G, Pontieri FE, Di Chiara G (1997). Cannabinoid and heroin activation of mesolimbic dopamine transmission by a common mu1 opioid receptor mechanism. Science.

[67] Scavone JL, Sterling RC, Van Bockstaele EJ (2013). Cannabinoid and opioid interactions: implications for opiate dependence and withdrawal. Neuroscience.

[68] Ashton CH (2001). Pharmacology and effects of cannabis: a brief review. Br J Psychiatry: J Mental Sci.

[69] Abrams DI, Couey P, Shade SB, Kelly ME, Benowitz NL (2011). Cannabinoid-opioid interaction in chronic pain. Clin Pharmacol Ther.

[70] Narang S, Gibson D, Wasan AD, Ross EL, Michna E, Nedeljkovic SS (2008). Efficacy of dronabinol as an adjuvant treatment for chronic pain patients on opioid therapy. The journal of pain : official journal of the American Pain Society.

[71] DeBeck K, Kerr T, Li K, Fischer B, Buxton J, Montaner J, et al. Smoking of crack cocaine as a risk factor for HIV infection among people who use injection drugs. CMAJ: Can Med Assoc J = journal de l'Association medicale canadienne. 2009;181(9):585–9. 10.1503/cmaj.082054 PMC2764753 19841052

[72] Falck RS, Wang J, Siegal HA, Carlson RG (2004). The prevalence of psychiatric disorder among a community sample of crack cocaine users: an exploratory study with practical implications. J Nerv Ment Dis.

[73] Darke S, Hall W (2003). Heroin overdose: research and evidence-based intervention. J Urban Health: Bull N Y Acad Med.

[74] Volkow ND, Baler RD, Compton WM, Weiss SR (2014). Adverse health effects of marijuana use. N Engl J Med.

[75] van Boekel LC, Brouwers EP, van Weeghel J, Garretsen HF (2013). Stigma among health professionals towards patients with substance use disorders and its consequences for healthcare delivery: systematic review. Drug Alcohol Depend.

[76] Syzdek MR, Green JD, Lindgren BR, Addis ME (2016). Pilot trial of gender-based motivational interviewing for increasing mental health service use in college men. Psychotherapy.

[77] McCoy CB, Lai S, Metsch LR, Messiah SE, Zhao W (2004). Injection drug use and crack cocaine smoking: independent and dual risk behaviors for HIV infection. Ann Epidemiol.

[78] Tyndall MW, Currie S, Spittal P, Li K, Wood E, O'Shaughnessy MV (2003). Intensive injection cocaine use as the primary risk factor in the Vancouver HIV-1 epidemic. AIDS.

[79] Degenhardt L, Singleton J, Calabria B, McLaren J, Kerr T, Mehta S (2011). Mortality among cocaine users: a systematic review of cohort studies. Drug Alcohol Depend.

[80] Lloyd-Smith E, Kerr T, Hogg RS, Li K, Montaner JS, Wood E (2005). Prevalence and correlates of abscesses among a cohort of injection drug users. Harm Reduct J.

[81] Bozinoff N, Wood E, Dong H, Richardson L, Kerr T, DeBeck K (2017). Syringe Sharing Among a Prospective Cohort of Street-Involved Youth: Implications for Needle Distribution Programs. AIDS Behav.

[82] Yamamoto A, Needleman J, Gelberg L, Kominski G, Shoptaw S, Tsugawa Y. Association between homelessness and opioid overdose and opioid-related hospital admissions/emergency department visits. Social science & medicine. 2019;242:112585. 10.1016/j.socscimed.2019.112585 PMC7023863 31634808

[83] Reid M (2020). A qualitative review of cannabis stigmas at the twilight of prohibition. J Cannabis Res.

[84] Darke S. Self-report among injecting drug users: a review. Drug and alcohol dependence. 1998;51(3):253–63; discussion 67–8. 10.1016/s0376-8716(98)00028-3 9787998

[85] Ahmad F, Jhajj AK, Stewart DE, Burghardt M, Bierman AS (2014). Single item measures of self-rated mental health: a scoping review. BMC Health Serv Res.

[86] Wang L, Panagiotoglou D, Min JE, DeBeck K, Milloy MJ, Kerr T (2016). Inability to access health and social services associated with mental health among people who inject drugs in a Canadian setting. Drug Alcohol Depend.

[87] Sarvet AL, Wall MM, Keyes KM, Olfson M, Cerda M, Hasin DS (2018). Self-medication of mood and anxiety disorders with marijuana: Higher in states with medical marijuana laws. Drug Alcohol Depend.

[88] Winkelman TNA, Admon LK, Jennings L, Shippee ND, Richardson CR, Bart G (2018). Evaluation of amphetamine-related hospitalizations and associated clinical outcomes and Costs in the United States. JAMA Netw Open..

